# Targeted approach to identify genetic loci associated with evolved dioxin tolerance in Atlantic Killifish (*Fundulus heteroclitus*)

**DOI:** 10.1186/1471-2148-14-7

**Published:** 2014-01-14

**Authors:** Dina A Proestou, Patrick Flight, Denise Champlin, Diane Nacci

**Affiliations:** 1 US Environmental Protection Agency, Office of Research and Development, National Health and Environmental Effects Research Laboratory, Atlantic Ecology Division, 27 Tarzwell Drive, Narragansett, RI 02882, USA; 2 Department of Ecology and Evolutionary Biology, Brown University, 80 Waterman Street, Providence, RI 02912, USA; 3 Current address: USDA Agricultural Research Service, 469 Center for Biotechnology and Life Sciences, 120 Flagg Road, Kingston, RI 02881, USA

**Keywords:** Adaptation, Ecotoxicology, Candidate gene scan, Killifish

## Abstract

**Background:**

The most toxic aromatic hydrocarbon pollutants are categorized as dioxin-like compounds (DLCs) to which extreme tolerance has evolved independently and contemporaneously in (at least) four populations of Atlantic killifish (*Fundulus heteroclitus*). Surprisingly, the magnitude and phenotype of DLC tolerance is similar among these killifish populations that have adapted to varied, but highly aromatic hydrocarbon-contaminated urban/industrialized estuaries of the US Atlantic coast. Multiple tolerant and neighboring sensitive killifish populations were compared with the expectation that genetic loci associated with DLC tolerance would be revealed.

**Results:**

Since the aryl hydrocarbon receptor (AHR) pathway partly or fully mediates DLC toxicity in vertebrates, single nucleotide polymorphisms (SNPs) from 42 genes associated with the AHR pathway were identified to serve as targeted markers. Wild fish (N = 36/37) from four highly tolerant killifish populations and four nearby sensitive populations were genotyped using 59 SNP markers. Similar to other killifish population genetic analyses, strong genetic differentiation among populations was detected, consistent with isolation by distance models. When DLC-sensitive populations were pooled and compared to pooled DLC-tolerant populations, multi-locus analyses did not distinguish the two groups. However, pairwise comparisons of nearby tolerant and sensitive populations revealed high differentiation among sensitive and tolerant populations at these specific loci: AHR 1 and 2, cathepsin Z, the cytochrome P450s (CYP1A and 3A30), and the NADH dehydrogenase subunits. In addition, significant shifts in minor allele frequency were observed at AHR2 and CYP1A loci across most sensitive/tolerant pairs, but only AHR2 exhibited shifts in the same direction across all pairs.

**Conclusions:**

The observed differences in allelic composition at the AHR2 and CYP1A SNP loci were identified as significant among paired sensitive/tolerant populations of Atlantic killifish with multiple statistical tests. The genetic patterns reported here lend support to the argument that AHR2 and CYP1A play a role in the adaptive response to extreme DLC contamination. Additional functional assays are required to isolate the exact mechanism of DLC tolerance.

## Background

Dioxin-like contaminants (DLCs), such as some polychlorinated biphenyls (PCBs), are highly toxic aromatic hydrocarbon pollutants whose ubiquitous occurrence presents global ecological and human health risks. The early life stages of fish are particularly sensitive to these toxic DLCs, and the Atlantic killifish, *Fundulus heteroclitus*, is one of the more sensitive fish species [1]. Despite this species’ relative sensitivity to DLC exposure, several wild killifish populations residing in heavily contaminated North American Atlantic coast estuaries have recently and independently evolved dramatic, heritable, and adaptive tolerance to DLCs [2], for which the mechanistic basis has yet to be fully explained. To address this issue, a targeted, candidate gene scan was performed to reveal genetic variation associated with tolerance in four wild DLC-adapted killifish populations.

Considerable effort has been spent attempting to identify the genetic and biochemical mechanisms underlying inter- and intra-specific variation in DLC sensitivity in vertebrates. Multiple lines of evidence support the crucial role of the aryl hydrocarbon receptor (AHR) pathway in DLC toxicity in mammals. Polymorphisms in the ligand binding domain of the AHR among mouse strains result in differences in ligand binding affinity, and low binding affinity appears to protect against all toxic responses to DLCs. In rats, DLC tolerance is associated with variation in the transactivation domain of the AHR, yet functional consequences of the variation are less predictable [3]. A candidate gene approach effectively identified two amino acid substitutions in the AHR among avian wildlife and a consistent relationship between the amino acid residues present and DLC sensitivity was observed at the species level [4,5]. In fish, the striking difference in DLC sensitivity among wild Atlantic tomcod populations has been attributed to a six base pair deletion in the AHR2 gene that results in a five-fold decrease in ligand binding affinity and reduced ability to promote expression of detoxification enzymes targeted by the AHR pathway [6].

Within the killifish AHR pathway, several non-synonymous single nucleotide polymorphisms (SNPs) have been identified in two AH receptor genes (AHR1 and AHR2), but patterns of genetic variation at these loci do not unequivocally reflect differences in DLC sensitivity among populations and no functional consequences (i.e., ligand binding and the ability to interact with xenobiotic metabolizing enzymes) were associated with AHR1 variants [7-9]. As an alternative to the candidate gene approach, Williams and Oleksiak [10,11] performed whole genome scans, whereby patterns of variation across hundreds of genetic loci were contrasted between killifish populations from polluted and reference sites in order to identify genes under selection with respect to DLC contamination. A handful of selectively important genetic markers were identified in each of three separate comparisons between populations residing in polluted habitats and their respective reference populations, and a single marker (in the CYP1A promoter) was found to be selectively important in all comparisons.

Both the single candidate gene approach and genome-wide scans (typically with sets of anonymous genetic markers) have led to great success in elucidating the genetic basis for many adaptive phenotypes [12,13], but neither has offered a comprehensive link to the observed variation in DLC sensitivity among wild killifish populations. A ‘candidate gene scan’ approach, which targets a relatively large set of expressed genes with known physiological function, should increase the probability of isolating genes that are under selection and relevant with respect to traits of interest [14]. Thus, this approach was adopted to maximize efficiency in the identification of genes associated with DLC tolerance, and complement previous and ongoing work investigating the mechanism(s) involved in the repeated adaptation to DLCs in wild killifish populations.

In this study, patterns of genetic variation at SNP markers distributed across genes that are components of, or whose expression is affected by the AHR and interacting pathways were examined among eight killifish populations that vary genetically in their responsiveness to DLCs. Four relatively uncontaminated populations whose sensitivities to a prototypical DLC (3,3’,4,4’,5- pentachlorobiphenyl, PCB126) range from 20–38 ng/L (reviewed in [12]) were chosen as sensitive populations. Each of these populations is located near one of four EPA-designated urban/industrial estuarine Superfund sites. Killifish resident to these sites are dramatically tolerant to the effects of PCB126, displaying LC20 values ranging from ~400 to ~ 8000 times higher than those for the sensitive killifish (Table 1). A companion study [9] provides a fine-scaled examination of genetic variation in three AHR-related loci (AHR1, AHR2, and AHRR) among killifish populations residing in uncontaminated and polluted habitats of the North Atlantic coast of the US, including some of the same populations examined in this study.

**Table 1:** Killifish population locations and site characteristics

**Site ID**	**Site location**	**Latitude × Longitude (W)**	**Distance from NBH, latitude, km**	**Latitudinal distance between pairs, km**	**LC20 (log), ng PCB126/L**	**DLC responsiveness**
NBH	New Bedford, MA	41.6676 × 70.9159	0	56	4.6	Tolerant
BI	Block Island, RI	41.1818 × 71.5793	56		1.4	Sensitive
BP	Bridgeport, CT	41.1570 × 73.2189	63	22	4.1	Tolerant
FLAX	Flax Pond, NY	40.9637 × 73.1342	85		1.6	Sensitive
NWK	Newark, NJ	40.7006 × 74.1223	115	26	4.1	Tolerant
SH	Sandy Hook, NJ	40.4687 × 74.0113	141		1.3	Sensitive
ER	Portsmouth, VA	36.8078 × 76.2945	547	55	5.3	Tolerant
KC	Gloucester, VA	37.3016 × 76.4226	492		1.4	Sensitive

Taken from Nacci et al. 2010. LC20 values represent the concentration of PCB126 resulting in 20% lethality of killifish embryos.

The question of whether the genetic variants observed at the targeted SNP markers in this study could explain the stark phenotypic differences between DLC-adapted (−tolerant) and -sensitive killifish populations was addressed in several ways. Measures of genetic diversity were compared between DLC-sensitive and DLC- tolerant groups and patterns of genetic differentiation among populations were tested against expectations of isolation by distance. In addition, by contrasting the behavior of individual loci among DLC-sensitive and DLC-tolerant populations, specific loci under selection were identified.

## Methods

### SNP marker development and preliminary screening

An extensive literature search was conducted to identify genes and biochemical pathways with demonstrated and potential involvement in the toxic responses to DLCs. A list of over 150 genes was compiled, which included components of the AHR pathway, nuclear receptors known to ‘cross talk’ with the AHR pathway (i.e., estrogen receptors, retinoic acid receptors, hypoxia inducible factors), cytochrome p450s, genes involved in cardiac development, cathepsins, and genes having oxidoreductase activity (e.g. [15], and references therein). The gene list was filtered at several stages of the marker development process. Sequence information for many, but not all, of the genes listed in Additional file 1 was retrieved from the *F. heteroclitus* unigene database in GenBank (http://www.ncib.nlm.nih.go/GenBank/). Additional unpublished sequences were kindly provided by Sibel Karchner (personal communication). Putative SNPs were detected with the QualitySNP pipeline [16]. In QualitySNP, sequences with > 95% similarity were assembled into contiguous sequences (contigs) using the sequence assembly program CAP3. Available contigs, those containing ≥ 4 overlapping sequences, were then evaluated for polymorphisms. Available contigs with SNPs were subjected to further scrutiny to assess the reliability of the putative SNPs identified. Specifically, SNPs were considered ‘true’ if they were represented in ≥ 2 ESTs, paralogous sequences within the cluster could be distinguished and haplotypes identified, and they were located in high quality sequence regions [16]. PCR primers and melting temperature (T_m_)-shift genotyping assays [17] were then designed for the ‘true’ SNPs with suitable flanking sequence (e.g. no SNPs, low sequence complimentarity in priming sites). A test panel of eight killifish from two populations (BI and NBH) was genotyped at 128 of the loci deemed highly reliable by QualitySNP. A SNP maker was considered valid if amplification product of the appropriate size was generated and polymorphism was observed among the eight individuals in the test panel. Outcomes for each stage of the SNP marker development process are detailed in Additional file 1.

### Fish collection

**Figure 1:**
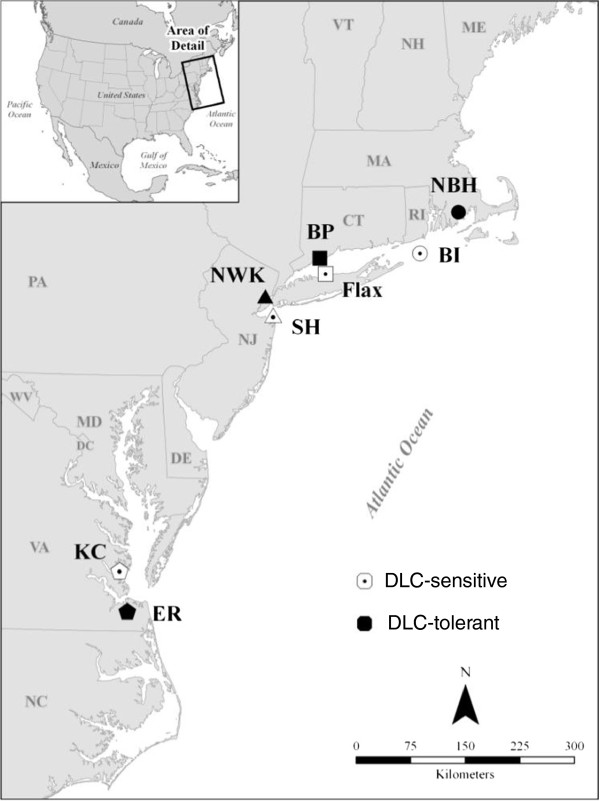
**Map of *Fundulus heteroclitus *** **collection sites along the Eastern U.S. coast.**

During the summer and fall months between 2008 and 2011, adult *Fundulus heteroclitus* were collected using baited minnow traps from eight estuarine sites spanning approximately 600 km of the Atlantic Coast of the USA (Figure 1). These specific killifish populations had already been characterized as either DLC-tolerant or DLC-sensitive, based on early life stage sensitivity to PCB126 [2,18,19]. To better assess genetic differences between tolerant and sensitive populations absent geographic influences, each DLC-tolerant population was paired with a nearby DLC-sensitive population. These pairs are located within the same or adjacent states but are separated by discontinuous shoreline and deep channels that limit fish migration and contaminant spread. Latitudinal distances between killifish residence sites (Table 1) were used to provide a consistent proxy to assess regional influences but were not intended to convey ‘as-the-fish-swims’ distances. Sixty to 100 live or sacrificed/frozen fish from each population were transported to the US Environmental Protection Agency (EPA) laboratory in Narragansett, RI, USA. Those that were transported live were sacrificed immediately upon return to the laboratory. Whole fish were stored at either −20 or −80°C prior to DNA extraction.

### Sample preparation and population genotyping

Genomic DNA was extracted from approximately 20 mg of caudal fin tissue from 36 or 37 of the 60–100 archived individuals per population according to the QIAGEN DNeasy protocol for animal tissue (optional RNase treatment included), quantified with the PicoGreen dsDNA assay (Invitrogen), and diluted to a standard concentration of 20 ng/μl. Diluted DNA extracts were submitted to the University of Minnesota’s BioMedical Genomics Center in a 96-well format for sample quality assessment and SNP genotyping using the Sequenom MassARRAY® technology. Three multiplex assays (containing 32, 27, and 12 SNPs respectively) were designed using MassARRAY® Designer software. Sampled *F. heteroclitus* for which DNA was extracted were genotyped with the first two plexes (because all SNP containing genes were represented) following the iPLEX assay protocol. Genotypes for each individual at each locus were called using the Sequenom System Typer Analysis package.

### Data analysis

Routine population genetics analyses were conducted using the freely available software package Arlequin ver. 3.5 [20]. Standard diversity indices, including the percentage of polymorphic loci (P_O_), average observed and expected heterozygosities (H_O_ and H_E_), and the within population fixation index (F_IS_), were determined for each sampled population. Indices were based on data from loci with < 10% missing data. Loci were also tested for departures from Hardy-Weinberg equilibrium (HWE) with 100,000 permutations and the percentage of loci in HWE was calculated for each population. The ability of this suite of SNP markers to detect genetic differentiation among populations was assessed by computing pairwise multi-locus F_ST_ estimates. To test whether the assumption of independence among loci would be violated by including multiple SNPs per gene in the analysis, data from a subset of SNPs (representing one SNP per gene) were also analyzed. Results did not differ significantly between the complete and limited datasets; therefore, only results from the complete dataset are reported here.

Genetic structure, where populations were assigned to groups defined by DLC sensitivity, was also included in the analysis and the pairwise multi-locus F_ST_ between the two groups was estimated with the ‘Compute pairwise F_ST_’ option in Arlequin ver. 3.5. An analysis of molecular variance (AMOVA) was performed to better understand how the observed genetic variance was partitioned within populations, among populations within each group, and between groups. In addition, as in [9], Student’s T-tests were used to detect significant differences in genetic diversity measures (P_O_ and H_O_) among the two groups.

For species like *F. heteroclitus* that are non-migratory, genetic differences at neutral loci should accumulate over time and generate a pattern of isolation by distance (IBD), whereby differentiation among populations increases with geographic distance [21]. Deviations from IBD patterns can be attributed to responses to local selection pressures [22]. Mantel tests and reduced major axis (RMA) regression analyses were performed in Isolation By Distance Web Service (IBDWS) [23] with 10,000 permutations to test for significant correlations between genetic distance (pairwise multi-locus F_ST_ values) and latitudinal distance. Mantel tests were also used to test for significant correlations between pairwise genetic differences and relative differences in sensitivity to PCB126 (LC20 as reported in [2]); moreover, partial Mantel tests were conducted to determine if there was a significant relationship between sensitivity to PCB126 and genetic divergence after taking latitudinal distance into account.

Although most of the genes included in this analysis were chosen based on prior work suggesting they might be responsive to DLCs, whether any or all contribute to the adaptive phenotype remains largely unresolved. To identify selectively important SNPs among the genes surveyed, F_ST_ values were calculated at each locus separately for each sensitive/tolerant comparison with the AMOVA function in Arlequin ver. 3.5. The statistical significance of each F_ST_ was determined through permutation testing with 10,000 iterations. An F_ST_ modeling approach similar to that described in [24] as implemented in Arlequin ver. 3.5 was also used to detect outliers. F_ST_ distributions conforming to a neutral model were simulated with 20,000 iterations, heterozygosities computed from empirical data, and assuming 10 demes. SNPs with F_ST_ values exceeding the 95th percentile of the null distribution were considered to be strong candidates for natural selection. In addition, as in [12], minor allele frequencies were calculated at each locus for each population with the expectation that loci associated with the adaptive phenotype would display shifts in allele frequency for each sensitive/tolerant pair. Consistent shifts in the same direction across all four comparisons were considered further evidence for selection acting at the loci.

## Results

### SNP markers

Killifish sequences representing 125 of the 150 candidate genes were mined from the GenBank nucleotide and unigene databases. CAP3 assembled those sequences into 183 contigs, of which 105 were available for further analysis (met the criterion of containing four or more overlapping sequences). Approximately 500 highly reliable putative SNP loci distributed among 50 genes were identified with the QualitySNP pipeline. T_m_-shift genotyping assays were designed for 128 of the highly reliable putative SNPs and the validity of each locus was tested by genotyping eight killifish collected from two populations (BI and NBH) (Additional file 1). Twenty-four of the 128 putative SNPs assayed (19%) did not amplify or produced uninterpretable melting curves. An additional 29 of the SNPs tested (23%) appeared to be monomorphic. Ultimately, 75 (59%) of the putative SNPs screened were polymorphic and 59 (those represented in the first two iplex assays) were used in the population genetic analysis (Table 2).

**Table 2:** List of SNP containing candidate genes included in the population genetic analysis

**Gene name**	**Unigene ID**	**Putative function**	**Reference**
Aryl hydrocarbon receptor 1	1743961[uid]	transcription factor, response to xenobiotic stimulus	[7]
Aryl hydrocarbon receptor 2	1743966[uid]	transcription factor, response to xenobiotic stimulus	[8]
Aryl hydrocarbon receptor 2b	N/A	transcription factor, response to xenobiotic stimulus	Karchner & Hahn, unpublished;
Aryl hydrocarbon receptor repressor	1743962[uid]	signal transducer, response to xenobiotic stimulus	[25]
Atrial natriuretic peptide	1743159[uid]	receptor binding, cardiac muscle hypertrophy in response to stress	[26]
Aldehyde dehydrogenase family 9 member A1	1741825[uid]	oxidoreductase activity, retinoic acid metabolic process	[27]
Cardiac myosin light chain-1	1741781[uid]	calcium ion binding, cardiac muscle tissue development	
Cathepsin E precursor	1744072[uid]	endopeptidase, antigen processing	
Cathepsin F precursor	1741927[uid]	cysteine-type peptidase activity	
Cathepsin Z precursor	2476770[uid]	cysteine-type peptidase activity, angiogenesis	
Complement Component C3	1742328[uid]	protein binding, lipid binding, endopeptidase inhibitor, innate immune response, regulation of angiogenesis	[28]
Cytochrome p450 1A	2476796[uid]	oxidoreductase activity, dibenzo-p-dioxin catabolic process	[11,29-31]
Cytochrome p450 3A30	1742582[uid]	oxidoreductase activity, response to xenobiotic stimulus	[28]
Cytochrome B5	1743444[uid]	enzyme binding, heme binding, oxidation reduction process	[29-31]
Estrogen Receptor alpha	1743972[uid]	estrogen receptor activity, transcription factor binding, response to estradiol stimulus, regulation of retinoic acid receptor signalling pathway	[32]
Estrogen Receptor beta a	2301213[uid]	estrogen receptor activity, estrogen response element binding, response to estradiol stimulus, regulation of transcription factor activity	[32]
Glyceraldehyde 3 phosphate dehydrogenase	1742899[uid]	oxidation reduction process	
Hepcidin	1744101[uid]	hormone activity, defense response	
Hepcidin 2	N/A		Karchner & Hahn, unpublished
Heatshock protein 90 beta	1743580[uid]	protein binding, response to stress	
Plasma Kallikrein precursor	1742469[uid]	peptidase activity, proteolysis, hemostasis	
Kallikrein	N/A		Karchner & Hahn, unpublished
Myosin Light chain 2	1743984[uid]	actin monomer binding, heart development	[29]
Myosin Light chain alkali, smooth muscle	1743182[uid]	structural constituent of muscle, muscle contraction	
NADH [ubiquinone] dehydrogenase 1 alpha subcomplex subuint 4	1743852[uid]	electron transport chain	[28]
Myosin regulatory light chain 3	1741657[uid]	calcium ion binding	
NADH dehydrogenase subunit 1	3014035[uid]	oxidoreductase activity	
NADH dehydrogenase [ubiquinone] 1 beta subcomplex subunit 10	1743800[uid]	electron transport chain	[33]
NADH dehydrogenase subunit 2	1742301[uid]	oxidoreductase activity	
NADH dehydrogenase subunit 3	1742764[uid]	oxidoreductase activity	
NADH dehydrogenase subunit 4	1741822[uid]	oxidoreductase activity	
NADH dehydrogenase subunit 6	3014152[uid]	oxidoreductase activity	
Platelet Coagulation Factor XI	3014242[uid]	peptidase activity, proteolysis, hemostasis	[28]
Retinoic Acid Receptor Responder Protein 1	1743266[uid]	negative regulation of cell proliferation	
Retinoic Acid Receptor Responder Protein 3	1741754[uid]	negative regulation of cell proliferation	
Rho-class Glutathione S-Transferase	1743333[uid]	transferase activity	[30,31,34]
Serine Protease Inhibitor	N/A	negative regulation of endopeptidase activity	Karchner & Hahn, unpublished
TBT Binding Protein	1741555[uid]	response to xenobiotic stimulus	[28]
Thioredoxin	1743303[uid]	protein binding, oxidoreductase activity	[33]
Translation Initiation Factor 2	1744423[uid]	ligand-dependent nuclear receptor binding, estrogen receptor binding, retinoic acid receptor binding, signal transduction	
Telethonin (Titin cap protein)	1743784[uid]	heart development, cardiac muscle contraction, sarcomere organization, response to stress	[29]
Cardiac Troponin T2	1742146[uid]	actin binding, tropomyosin binding, troponin binding, cardiac muscle morphogenesis, sarcomere organization, muscle filament sliding	[26,29,35]
Ubiquitin A-52 residue ribosomal protein fusion product 1	1744070[uid]	structural constituent of ribosome, translation	

Note: Potential involvement of each gene in toxic DLC response was demonstrated in citations listed in ‘Reference’ column.

N/A = not available.

### Standard diversity indices

Substantial genetic variation was observed in each killifish population examined. The percentage of polymorphic loci (P_O_) ranged from 51% to 73% across populations, with a greater percentage of monomorphic loci in NBH and the Virginia populations. A similar pattern was detected when observed heterozygosity (H_O_) was used to measure diversity; ER, KC and NBH populations were the least heterozygous. According to the average within population fixation index (F_IS_), the loss of heterozygosity in NBH, ER, KC, and SH was statistically significant (Table 3).

**Table 3:** **Genetic parameters for sampled *F. heteroclitus *** **populations**

**Population**	**P**_**O**_	**H**_**O**_	**H**_**E**_	**% HWE**	**F**_**IS**_	**MAF**
BI	65.31 (49)	0.15	0.15	71.88	0.042	0.16
NBH	53.19 (47)	0.14	0.16	76.00	0.171*	0.20
BP	64.58 (48)	0.15	0.15	93.55	0.001	0.16
FLAX	61.22 (49)	0.16	0.17	90.00	0.059	0.19
SH	73.33 (45)	0.16	0.21	78.79	0.240*	0.19
NWK	64.58 (48)	0.15	0.16	80.65	0.031	0.19
ER	51.02 (49)	0.12	0.15	60.00	0.108*	0.21
KC	57.14 (49)	0.14	0.17	82.14	0.122*	0.22
Mean	61.30	0.15	0.17	79.12	0.097	0.19
Standard Deviation	7.28	0.01	0.02	10.46	0.080	0.02

P_O_ = number of polymorphic loci/number of usable loci (in parentheses); H_O_ = sum of observed heterozygosity for each usable locus/number of usable loci; H_E_ = sum of expected heterozygosity for each usable locus/number of usable loci; % HWE = number of loci in Hardy-Weinberg equilibrium/number of polymorphic loci; F_IS_ = population specific fixation index; MAF = average Minor Allele Frequency, calculated for polymorphic loci only. *p ≤ 0.05 based on 10,000 permutations.

A majority of the 59 SNP loci assayed (64%) were considered to be common SNPs, with minor allele frequencies greater than 0.1, when data from all populations were pooled. For each population, the percentage of common SNPs ranged from 54% to 76% with BP and ER at the two extremes. Within each population the minor allele frequency, averaged across all polymorphic loci, varied between 0.16 and 0.22. The mean minor allele frequency was lowest in BP and BI fish and highest in ER fish (Table 3).

Most of the SNP loci in this study were found to be in Hardy-Weinberg equilibrium within each population. Again, the population with the fewest loci conforming to Hardy-Weinberg expectations was ER (Table 3). In total, 14 loci deviated from HWE, but no single deviation was consistent across all eight populations or across the four tolerant populations.

### Genetic differentiation

**Figure 2:**
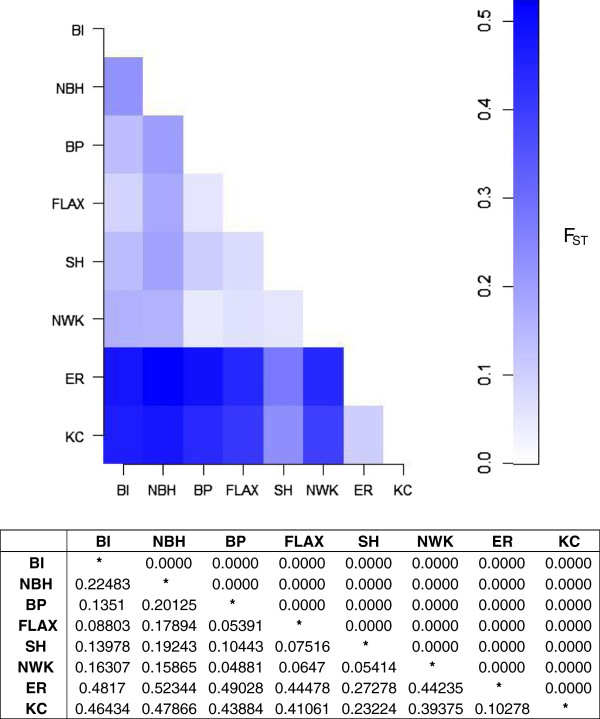
**Pairwise F**_**ST **_**comparisons demonstrating genetic differences between all populations.** F_ST_s calculated with data from all SNP loci. Values below the diagonal represent genetic distances and values above the diagonal represent significance level. BI = Block Island, RI, NBH = New Bedford Harbor, MA, BP = Bridgeport, CT, FLAX = Flax Pond, NY, SH = Sandy Hook, NJ, NWK = Newark, NJ, ER = Elizabeth River, VA, KC = Kings Creek, VA.

Mean pairwise F_ST_ values for all population comparisons were found to be statistically significant and suggest moderate to very high levels of genetic differentiation. Overall, markedly higher genetic differentiation was detected between the two Virginia populations and all others (Figure 2). A standard analysis of molecular variance (AMOVA) confirmed that a substantial proportion (23%) of the observed molecular variation can be attributed to differences among populations.

No significant differentiation emerged when populations were grouped by DLC sensitivity. The expectation that, on average, DLC-adapted populations are less diverse (as quantified by P_O_ and H_O_) than DLC-sensitive populations, was not supported (Table 4). The genetic differentiation between the two groups as measured by F_ST_ was 0.0212; however, the hierarchical AMOVA results suggest that the groups were not significantly different (Table 5).

**Table 4:** T-test results

**Diversity measure**	**DLC-sensitive**	**DLC-tolerant**	**P-value**
P_O_	0.64 (0.07)	0.58 (0.07)	0.28
H_O_	0.15 (0.01)	0.14 (0.01)	0.23
H_E_	0.18 (0.03)	0.16 (0.01)	0.18
MAF	0.15 (0.02)	0.15 (0.01)	1.0

Comparison of mean genetic diversity measures between DLC-sensitive and DLC-tolerant *F. heteroclitus* populations. Standard deviations are in parentheses. P_O_ = average percentage of polymorphic loci within groups; H_O_ = average observed heterozygosity within groups; H_E_ = average expected heterozygosity within groups; MAF = average minor allele frequency within groups. Results are based on genotype information gathered from 36/37 individuals per population at 59 SNP loci representing 42 genes.

**Table 5:** AMOVA results (populations grouped according to DLC sensitivity); data from all loci, CYP1A, and AHR2

**Source of variation**	**df**	**Sum of squares**	**Variance components**	**Percent variation**
**All Loci**				
Among Groups	1	31.573	−0.19211 (V_a_)	−4.38
Among Pops w/in Groups	6	528.228	1.15127 (V_b_)	26.27
W/in Pops	580	1985.789	3.42377 (V_c_)	78.12
Total	587	2545.590	4.38293	
**CYP1A**				
Among Groups	1	2.726	−0.01613 (V_a_)	−6.52
Among Pops w/in Groups	6	44.501	0.09971 (V_b_)	40.32
W/in Pops	574	93.977	0.16372 (V_c_)	66.20
Total	581	141.204	0.24730	
**AHR2**				
Among Groups	1	12.822	0.03671 (V_a_)	13.50
Among Pops w/in Groups	6	13.271	0.02775 (V_b_)	10.20
W/in Pops	570	118.296	0.20754 (V_c_)	76.30
Total	577	144.389	0.27200	

### Isolation by distance

**Figure 3:**
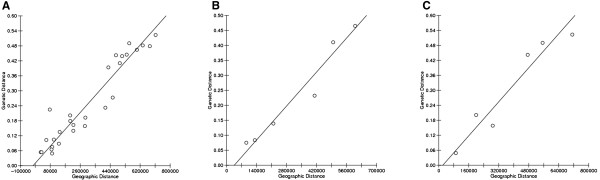
**Reduced major axis regression of genetic distance and geographic distance.** Genetic distance = pairwise F_ST_; Geographic (latitudinal) distance in meters. **(A)** All populations **(B)** DLC-sensitive populations only **(C)** DLC-tolerant populations only.

A regression of the genetic and latitudinal distance matrices when all populations were included in the analysis was statistically significant (r =0.8607, p < 0.001) reflecting a pattern of IBD (Figure 3A). Given that populations were sampled from three distinct geographic regions differentially impacted by the Pleistocene glacial retreat, the observed relationship is most likely driven by long-term history and demography rather than contemporary forces [22]. Not surprisingly, the pattern persisted when only sensitive populations were considered (r = 0.9223, p = 0.0372). A clear positive relationship was also apparent when only tolerant populations were included in the analysis (r = 0.9443), but the trend was not significant (p = 0.0856) (Figure 3C). The lack of a significant IBD pattern among DLC-adapted populations could be indicative of local selection (in response to chemical contamination) counteracting the effects of history, demography, migration, and drift, but is more likely a consequence of small sample size. It was hypothesized that if selection is a major force shaping patterns of genetic variation among the sampled killifish populations, a strong correlation between pairwise genetic differences and relative differences in sensitivity to PCB126 should be evident. Additional Mantel tests found no such relationship (data not shown).

### Loci under selection

The number of loci with significant F_ST_ values greater than 0.10, suggesting they are greatly differentiated among populations [36], varied among the four sensitive/tolerant population pairings. Of the four comparisons, the largest number of highly differentiated loci was detected between BI and NBH (Table 6). Included among these loci were the aryl hydrocarbon receptors (AHR) 1 and 2, cathepsin Z, the cytochrome P450s (CYP 1A and 3A30), and the NADH ubiquinone oxidoreductase MLRQ subunit. Only the CYP1A locus exhibited a consistently high F_ST_ value across all population pairs while both AHR genes were highly differentiated in all comparisons except for that between SH and NWK. While great differences were detected between paired populations at the CYP1A locus, a hierarchical AMOVA did not confirm that those differences are associated with DLC sensitivity (Table 5). In contrast, variation at the AHR2 locus could distinguish DLC-sensitive and tolerant groups (Table 5).

**Table 6:** **Single locus F**_**ST **_**values demonstrating high differentiation among population pairs**

	**Population comparison**			
**Locus**	**BI/NBH**	**FLAX/BP**	**SH/NWK**	**KC/ER**
Aryl hydrocarbon receptor 1_1530	**0.48393**	**0.15677**	−0.00349	**0.12191**
Aryl hydrocarbon receptor 1_161	**0.26498**	0.00028	0.00929	N/A
Aryl hydrocarbon receptor 1_2289	**0.21749**	N/A	−0.00376	N/A
Aryl hydrocarbon receptor 1_948	**0.35061**	−0.0137	−0.01388	N/A
Aryl hydrocarbon receptor 2_1929	**0.25477**	**0.1825**	0.04692	**0.19729**
Aryl hydrocarbon receptor 2_792	**0.36916**	N/A	0.00417	N/A
Cathepsin F_653	**0.18941**	−0.0137	−0.01271	0.03401
Cathepsin Z_624	**0.22978**	0.04241	−0.00759	−0.00325
Cytochrome P450 1A_2140	**0.37453**	**0.3733**	**0.20304**	**0.70083**
Cytochrome P450 3A_1166	**0.51614**	−0.01332	−0.00179	−0.01449
Hepcidin2_399	**0.14131**	0.06457	0.00719	0.04197
Heat shock protein 90_775	0.09438	N/A	0.02932	**0.12676**
NADH ubiquinone oxidoreductase MLRQ subunit_363	**0.32115**	−0.01268	**0.12679**	−0.01516
Myosin regulatory light chain 3_372	**0.11663**	−0.00843	−0.01529	0.0674
NADH dehydrogenase subunit 4_347	0.0137	N/A	**0.24148**	N/A
NADH dehydrogenase subunit 4_586	0.01315	N/A	**0.13504**	0.01408
NADH dehydrogenase subunit 4_669	0.01259	N/A	**0.25714**	N/A
NADH dehydrogenase subunit 6_787	0.01486	N/A	**0.26028**	N/A
Serine protease inhibitor_938	0.00078	N/A	0.03176	**0.10229**
TBT binding protein_635	**0.10673**	−0.00786	−0.00228	N/A
Thioredoxin_582	**0.19931**	0.01625	−0.00785	0.1248

F_ST_ values for highly differentiated loci across all four DLC-sensitive/-tolerant population pairs. F_ST_s were calculated using the locus by locus AMOVA function in Arlequin v. 3.5. Values exceeding an F_ST_ threshold of 0.1 are noted in bold and are significant at p < 0.05. Comparisons that were not made because the markers were monomorphic in some populations are noted as N/A. BI = Block Island, NBH = New Bedford Harbor, Flax = Flax Pond, BP = Bridgeport, SH = Sandy Hook, NWK = Newark, KC = Kings Creek, and ER = Elizabeth River.

**Figure 4:**
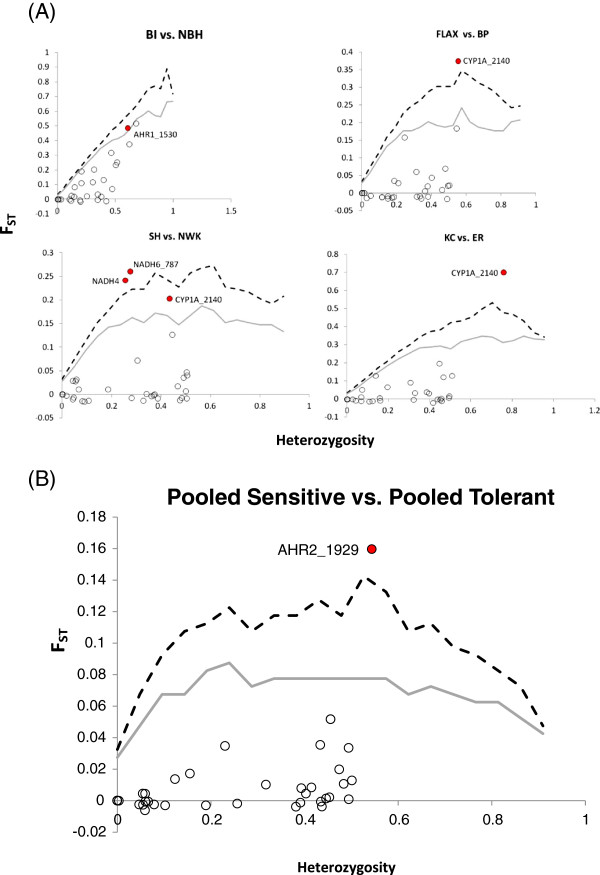
**Results of an F**_**ST **_**modeling approach to detect loci under selection.** Empirical F_ST_ values have been plotted against heterozygosity for each pairing of DLC-sensitive and DLC-tolerant sites **(A)** and for the pooled DLC-sensitive/-tolerant comparison **(B)**. Gray lines represent the 95^th^ percentile and black, dotted lines represent the 99^th^ percentile. Red data points indicate outlier SNPs with respect to the F_ST_ null distribution.

Further exploration of associations between specific loci and DLC sensitivity using an F_ST_ modeling approach produced results similar to those derived from the locus-by-locus AMOVA. Again, the CYP1A locus was identified as a significant outlier with respect to the simulated F_ST_ null distribution in three out of the four pairwise comparisons between each sensitive population and its DLC-tolerant counterpart. Moreover, when data for all sensitive populations were pooled and compared to the pooled data for all tolerant populations, the AHR2 locus emerged as a significant outlier (Figure 4). These findings lend support to the hypothesis that the CYP1A and AHR2 loci may be involved in the evolution of DLC tolerance.

**Figure 5:**
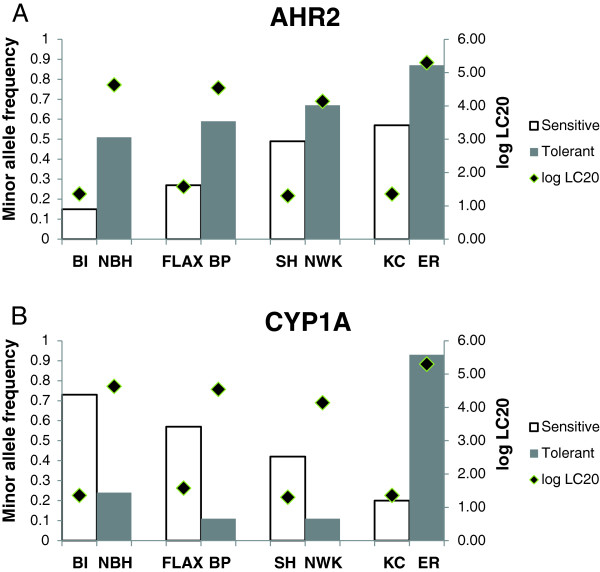
**Shifts in minor allele frequencies across populations for (A) AHR2 and (B) CYP1A.** Populations are arranged with respect to geographical region. Corresponding log LC20 values measured in response to PCB126 for each population (Nacci *et al*. 2010) are also noted.

Evidence of selection can also be gleaned from subtle shifts in allele frequencies in response to environmental variables after controlling for population structure [37]. By reviewing differences in minor allele frequency between sensitive and DLC-adapted population pairs, a strong signal was apparent for two SNPs: AHR2_1929 and CYP1A_2140 (Table 7). Upon further examination, the shift in minor allele frequency for the AHR2 SNP varied in magnitude but was consistently in the same direction for all four population comparisons, providing yet another line of evidence linking AHR2 with the DLC-tolerant phenotype. This was not the case at the CYP1A locus. Although substantial differences in minor allele frequency were observed between each sensitive and tolerant pair, the frequency shift between KC and ER was in the direction opposite of the other three pairings (Figure 5).

**Table 7:** **Minor allele frequencies of *F. heteroclitus *** **SNP markers calculated for each population**

		**Population**							
**Locus**	**Minor allele**	**BI**	**NBH**	**FLAX**	**BP**	**SH**	**NWK**	**KC**	**ER**
Aglobin_89	A/**G**	0	0	0	0	0	0	0	0
AHR1_1530	C/**G**	0.08	0.62	0.23	0.03	0.26	0.2	0.44	0.19
AHR1_161	C/**G**	0.14	0.5	0.04	0.01	0.03	0.08	0	0
AHR1_2289	G/**T**	0.07	0.64	0	0	0.03	0.05	0	0
AHR1_948	C/**T**	0.07	0.49	0.01	0.01	0.03	0.03	0	0
**AHR2_1929**	**C**/T	0.15	0.51	0.27	0.59	0.49	0.67	0.57	0.87
AHR2_792	C/**T**	0.07	0.5	0	0	0.03	0.07	0	0
AHR2B_992	**A**/G	0	0	0.05	0.07	0.05	0.01	0	0
AHRR_1095	C/**T**	0	0	0.11	0.05	0	0	0	0.06
AHRR_1299	C/**T**	0	0	0.15	0.05	0	0	0.01	0.06
ANP_521	C/**T**	0.24	0.44	0.7	0.41	0.29	0.23	0.38	0.28
BAD_213	G/**T**	0.15	0.28	0.24	0.27	0.21	0.28	0.06	0.07
Bglobin_193	C/**T**	0	0	0	0.01	0.03	0.01	0.1	0.07
Cardiac_MLC1_250	C/**T**	0	0	0	0	0	0	0	0
CathE_730	A/**C**	0	0	0	0	0	0	0	0
CathF_653	A/**T**	0.2	0	0.09	0.09	0.21	0.2	0.14	0.26
CathZ_624	G/**T**	0.5	0.15	0.19	0.34	0.43	0.38	0.31	0.38
CC3_571	A/**G**	0	0	0	0	0	0	0	0
**CYP1A_2140**	**A/T**	0.73	0.24	0.57	0.11	0.42	0.11	0.2	0.93
CYP3A_1166	**A**/G	0.78	0.19	0.42	0.43	0.21	0.28	0.06	0.06
CytB5_626	**G**/T	0.01	0	0.01	0.01	0.06	0.05	0.07	0
ERa_1497	**G**/T	0	0	0	0	0	0	0	0
ERba_1373	**C**/T	0.19	0.25	0.51	0.38	0.68	0.47	0.04	0.03
GP3D_530	**A**/G	0	0	0	0.01	0	0	0	0
Hepcidin_69	C/**T**	0.11	0.08	0.07	0.16	0.01	0.03	0	0
Hepcidin2_399	A/**G**	0.12	0.37	0.26	0.11	0.39	0.49	0.68	0.82
HSP90_775	A/**G**	0.01	0.14	0	0	0.04	0	0.14	0
Kallikrein_502	G/**T**	0	0	0	0	0	0	0	0
KallikreinS_309	A/**G**	0	0	0	0	0	0	0	0
MLC2_436	**C**/T	0	0	0.07	0.05	0.14	0.07	0.19	0.33
MLC2_535	A/**C**	0	0	0.03	0.01	0.07	0	0.32	0.35
MLCA_577	**C**/T	0.09	0.03	0.08	0.08	0.26	0.09	0.03	0.01
MLRQ_363	A/**T**	0.46	0.07	0.11	0.09	0.42	0.17	0.7	0.7
MRCL3_372	**A**/G	0.27	0.14	0.22	0.18	0.05	0.04	0.17	0.04
NADH10_107	A/**T**	0.19	0.03	0.15	0.24	0.14	0.05	0.13	0.31
NADH10_439	**C**/T	0.31	0.28	0.23	0.19	0.25	0.18	0.51	0.64
NADH2_349	**A**/G	0	0	0.11	0.05	0	0.05	0.03	0
NADH3_165	C/**G**	0	0	0	0	0	0	0	0
NADH4_347	C/**T**	0.03	0	0	0	0.26	0	1	1
NADH4_586	A/**G**	0.03	0	0	0	0.27	0.05	0.03	0
NADH4_669	G/**T**	0.03	0	0	0	0.28	0	1	1
NADH6_787	**A**/G	0.08	0.05	0	0	0.28	0	1	1
PCFXI_511	**G**/T	0	0	0	0	0	0	0	0
RARR1_759	A/**C**	0.08	0.14	0.22	0.21	0.52	0.35	0.7	0.39
RGST_397	A/**T**	0.1	0.12	0.19	0.3	0.14	0.19	0.52	0.64
SPI_938	**A**/G	0.04	0.08	0	0	0.06	0	0.27	0.08
TBTbp_635	**A**/T	0.03	0.18	0.04	0.02	0.02	0	0	0
Thioredoxin_582	**A**/G	0.3	0.04	0.53	0.65	0.47	0.5	0.75	0.49
TIF2_727	**C**/T	0	0	0	0	0.04	0.04	0.11	0.06
Titin_478	**C**/G	0.19	0.1	0.46	0.26	0.42	0.3	0.66	0.64
TroponinT2_653	**C**/T	0.3	0.19	0.28	0.19	0.38	0.4	0.17	0.23

Zero values indicate populations where the SNP marker was monomorphic. Minor alleles in bold.

## Discussion

Four independent DLC-adapted killifish populations were contrasted with neighboring sensitive counterparts in an attempt to reveal genetic loci associated with intra-specific DLC tolerance in the wild. The AHR pathway is known to mediate toxic responses to DLCs in all vertebrates and several studies involving killifish have implicated altered AHR pathway function in eliciting the tolerant phenotype [29,30,37]. Therefore, a ‘candidate gene scan’ approach, focused on components and targets of the AHR and interacting pathways represented among available killifish genetic resources, was applied to gain a better understanding of the genetic basis for the observed phenotypic variation in DLC sensitivity among killifish populations.

Genetic diversity and population structure of tolerant killifish populations were examined with the expectation that patterns of historical stress would be revealed. Levels of genetic diversity and population structure in killifish have been evaluated in several studies; some with the specific aim of addressing the impacts of pollutant-driven selection [9,22,38-41]. Results of the multi-locus analyses conducted here are in agreement with previous reports. Comparably high levels of genetic variation were estimated for all populations, rendering the hypothesis that a genetic bottleneck facilitated the emergence of the DLC-adaptive phenotype highly unlikely. With respect to population structure, each population included in this study was moderately to highly different from all others and observed F_ST_ values were comparably higher than earlier measures for killifish populations spanning a similar geographic range (e.g. F_ST_ ranged from 0.03 - 0.2 in [38] and from 0.01 - 0.24 in [41]). The difference in the extent of population structure detected among independent studies could be a consequence of the type of markers used (targeted SNPs vs. putatively neutral microsatellites); however, the overall patterns are consistent: genetic differences appear to be driven by geographic distance (latitudinal or shoreline) rather than DLC sensitivity. Moreover, a hierarchical AMOVA did not attribute any of the existing molecular variation to differences between DLC-sensitive and DLC-tolerant groups.

The failure to detect a significant relationship between multi-locus measures of genetic diversity/differentiation and increased tolerance to DLCs may be because a majority of the markers used in the current and previous analyses are selectively neutral. It has been suggested that neutral markers best reflect the effects of anthropogenically-mediated environmental change when populations are in decline and genetic exchange among populations is restricted [42]. There is no evidence that either sensitive or DLC-tolerant killifish populations have experienced a reduction in population size [22,38,39]. A more plausible explanation for the rapid adaptation to DLC contamination is that the trait in question is controlled by a small number of loci [39,43]. This explanation is consistent with theoretical models that predict single allelic differences of large effect dominate adaptive shifts when environmental change is sudden and selection is intense [42]. The prediction has been tested and verified in insect populations reacting to pesticides [44], benthic marine invertebrates subject to heavy metal toxicity [45], and fish exposed to DLCs [6].

Phenotypic similarities among tolerant populations exposed to a prototypical DLC (e.g. LC20) [2] supported an expectation of common loci under selection; however, genetic loci associated with tolerance might vary across populations due to differences in the selective agents present in their native habitats. The presumed selective agents include large classes of aromatic hydrocarbons whose toxic effects are mediated fully (DLCs) or partly (polycyclic aromatic hydrocarbons, PAHs) through the AHR pathway. While urban contamination includes moderate levels of both PAHs and PCBs, toxic levels of DLCs have been measured at NBH and NWK, PAHs at ER, and both at BP [2,46]. Therefore, genetic analyses were conducted to identify common loci under selection in tolerant populations, and differences between sensitive/tolerant paired killifish populations.

Since functional variation in AHR-ligand binding initiates the AHR pathway cascade (and largely explains some intra- and inter-species differences in DLC sensitivity, e.g. [15] and references therein), evidence for selection acting on AHR loci was of obvious interest in comparing tolerant versus sensitive killifish populations. As in other fish species, killifish possess at least two distinct AH receptor genes, AHR1 and AHR2, and the expression of AHR2 predominates in most tissues [47]. Strong signals of selection were detected for an AHR1 (AHR1_1530) and AHR2 SNP (AHR2_1929) included in this analysis in three of the four pairwise comparisons (SH/NWK excluded) when locus-by-locus F_ST_ were considered. Although these two SNPs are synonymous and do not result in amino acid changes, they are both located in the transactivation domain of their respective AHR genes and in close proximity to non-synonymous SNPs found to be under selection in Reitzel et al. [9]. Minor allele frequencies (equivalent to base frequencies referred to in [9]) of the AHR2_1929 SNP were consistently higher in the DLC-tolerant populations for all four population pairs. Although the F_ST_ modeling approach identified the AHR1_1530 SNP as a significant outlier in only one of the comparisons (BI/NBH), when genotype data from all DLC-sensitive populations were pooled and compared to that of all DLC-tolerant populations AHR2_1929 was the only locus found to deviate from neutral expectations. Similar patterns of genetic variation in AHR1 and AHR2 loci is not surprising given that these two genes are arranged in tandem (and therefore linked) within the killifish genome [9,48]. To determine whether variation in both loci, a single locus, or neither locus underlies the DLC-tolerant phenotype, population genetic data must be accompanied by functional assays. A focused examination of allelic variation in killifish AHR1 and AHR2 revealed SNPs under positive selection in both genes; however, AHR1 variants were not responsible for alterations in receptor function and AHR2 variations have yet to be tested [7,9]. Studies investigating the genetic basis for DLC-tolerance in zebrafish and Atlantic tomcod have isolated an AHR2 gene as a key player in mediating DLC sensitivity [6,48]; while AHR1 and AHR2 seem to play functional roles in dioxin toxicity in red seabream [35]. Taken together, these results suggest that AHR2 variation likely plays a strong role in DLC sensitivity and tolerance in killifish, but more complex interactions may be revealed as new AHR paralogs are being identified and characterized [9].

Consistent with the refractory induction patterns for CYP1A (an early and sensitive marker of AHR pathway activation) observed among DLC-tolerant killifish populations in laboratory studies [2,29,49], the CYP1A SNP emerged as a strong candidate for selection in all tolerant populations. High F_ST_ values and large differences in minor allele frequencies were observed at the CYP1A locus in all four population comparisons and the F_ST_ modeling approach identified CYP1A as a significant outlier in three out of the four comparisons (BI/NBH excluded). However, the shift in allele frequency, although significant, was not in the same direction across all comparisons. A full genome scan analysis of three of the same tolerant killifish populations (excluding BP) and their sensitive counterparts also identified a SNP marker in the CYP1A promoter region as the only locus (out of 354 screened) under selection in all three DLC-tolerant populations surveyed, but again, the direction of the allele frequency shift between DLC-sensitive and tolerant populations was not uniform [11]. In a follow-up study, Williams and Oleksiak [50] found that CYP1A promoter variants derived from a DLC-tolerant population (NBH) resulted in elevated expression of CYP1A *in vitro*, contradicting the well-documented refractory response of CYP1A to DLC exposure among tolerant populations *in vivo*. These previously reported results, coupled with the knowledge that the CYP1A SNP included in this analysis is located in the 3’ untranslated region of the gene [51] make the exact functional role (if any) of CYP1A SNPs in the DLC-tolerant phenotype unclear. It may be that additional, yet to be discovered factors associated with CYP1A regulation are also important.

The interpretation of CYP1A as a candidate for selection must also take into account that tolerance has evolved in response to different AHR ligands. Specifically, the role of CYP1A in AHR-mediated toxicity varies by ligand class. Classically described, AHR agonists induce the production of enzymes (predominantly CYP1A) that metabolize PAHs, but not DLCs. Unlike DLC toxicity, PAH toxicity is self-limiting, due to AHR-enhanced PAH elimination, and includes components that are not AHR-mediated. For example, CYP1A knockdown studies in zebrafish embryos have demonstrated the protective value of CYP1A during developmental PAH exposures [52]. That tolerant killifish populations from varied selective environments (PAH- versus DLC-dominated pollution profiles) show similar, poorly-inducible CYP1A phenotypes warrants the consideration of the adaptive value of the CYP1A recalcitrant phenotype more broadly, e.g., as an energy conservation strategy associated with chronic pollution exposures. As in other species [53,54], variation in the CYP1A sequence among killifish populations may be beneficial if associated with conditional fitness under transient, low level PAH exposures. Alternatively, variation in CYP1A may be related to its position ‘downstream’ in the AHR pathway, and secondary to changes in ‘upstream’ loci, causally associated with tolerance. Regardless of mechanism, variation at the CYP1A locus differentiates tolerant from sensitive killifish.

Among nearby sensitive/tolerant killifish population comparisons, the BI/NBH pairing was the most genetically differentiated based on multi-locus F_ST_; many additional loci were identified as candidates for selection (i.e., F_ST_ > 0.1 and significant allele frequency shifts). In addition to the AHRs and CYP1A, these populations appear to be highly differentiated at cathepsin F, cathepsin Z, CYP3A30, and the NADH ubiquinone oxidoreductase MLRQ subunit loci. Cathepsins are a large group of proteolytic enzymes that have been implicated in cardiomyopathies and cardiovascular disease, ultimately resulting in impaired pump function [55,56]. Given that the cardiovascular system is a main target of DLC toxicity in all vertebrates [48], it is reasonable to propose that alterations in the cathepsin coding sequence could contribute to existing differences in DLC sensitivity. CYP3A30 is an abundant xenobiotic metabolizing enzyme in killifish livers responsible for the breakdown and clearance of a wide array of anthropogenically derived pollutants [57] and has been identified as a target of the AHR pathway [58]. In killifish, expression of this gene was found to be significantly higher in field caught ER females relative to those collected from KC [31]; however, it was not differentially expressed in killifish embryos derived from the same DLC-tolerant and sensitive populations included in this study when exposed to PCB126 under controlled laboratory conditions [29,37]. Mutations in the NADH ubiquinone oxidoreductase MLRQ subunit are associated with metabolic diseases [28]. Again, expression of this gene was found to be significantly higher in field caught ER females relative to those collected from KC [31]. Moreover, expression of another component of the NADH ubiquinone oxidoreductase enzyme, NDUB2, was found to be higher in the brains of NBH, NWK, and ER adult fish [59]. The repeated association of NADH ubiquinone oxidoreductase with DLC-tolerance suggests that biochemical pathways other than the AHR could be involved in this chemically-induced stress response.

It is not known whether the identification of additional candidate loci for strong selection in the BI/NBH pair only suggests unique tolerance-related or biologically-relevant differences (BI is the only oceanic rather than estuarine site included in this analysis) or technical artifacts (statistical power). While similar phenotypes among the four tolerant populations examined suggest a conserved biochemical basis for intra-specific DLC tolerance in killifish, whether that similarity is constrained to identical nucleotide changes remains to be seen. The genetic mechanisms of adaptation could vary among DLC-tolerant populations. Alternatively, the differences detected may be a reflection of additional unique stressors encountered by each population pair.

## Conclusions

The purpose of this study was to identify genetic polymorphisms associated with DLC sensitivity in Atlantic killifish from a suite of candidate loci involved in the AHR and interacting pathways; whether the polymorphisms in and of themselves are responsible for the drastic differences in DLC sensitivity among populations was beyond the scope of this work. Two loci, AHR2 and CYP1A, displayed patterns of variation consistent with selection in each of four pairwise comparisons. Additional loci with specific alleles significantly overrepresented in some, but not all, DLC tolerant populations underscore the possibility that the genetic variation in each population may have been shaped by similar yet unique selection pressures. Although the intention was to include all components of the AHR and interacting pathways in this population screen, the underrepresentation or absence of key genes (e.g., aryl hydrocarbon receptor nuclear translocator (ARNT)) in the *F. heteroclitus* unigene database resulted in an enriched but incomplete set of candidate genetic markers for DLC toxicity and (presumably) tolerance. Genetic resources being made available through the *Fundulus* genome consortium as well as a parallel, unbiased, Quantitative Trait Locus (QTL) approach to the discovery of the genetic mechanism of DLC tolerance in killifish will greatly increase our understanding of this dramatic example of anthropogenically induced, rapid adaptation in the wild.

### Availability of supporting data

The sequences for the SNPs analyzed in this article are available through the Dryad Digital Depository and can be accessed by searching for the data package title *Data from: Targeted approach to identify genetic loci associated with evolved dioxin tolerance in Atlantic Killifish* (Fundulus heteroclitus), doi:10.5061/dryad.2355t Data files: Proestou_etal_Killifish_SNP_sequences.

## Abbreviations

DLC: Dioxin-like compound; AHR: Aryl hydrocarbon receptor; PCB: Polychlorinated biphenyl; PAH: Polycyclic aromatic hydrocarbon; BI: Block Island; NBH: New bedford harbor; BP: Bridgeport; FLAX: Flax pond; SH: Sandy hook; NWK: Newark; ER: Elizabeth river; KC: Kings creek.

## Competing interests

The authors declare they have no competing interests.

## Authors’ contributions

DAP, PF, DC, and DN contributed to study design, sample collection and processing. DP performed data analysis. DP and DN wrote the manuscript. All authors read and approved the final manuscript.

## Supplementary Material

Additional file 1 **List of all genes considered for SNP analysis and their fate during the marker development process.** Sequences provided by Sibel Karchner were derived from either 26 (AHR1, AHR2, and AHRR) or 10 individuals (not cDNA libraries) collected from Scorton Creek, MA. In some cases, Qualitiy SNP identified multiple available contigs from a single unigene entry. The number of SNPs genotyped does not equal the number of SNPs in the analysis because 8 of the markers failed to provide quality genotype information. N = No, Y = Yes, N/A = Not available, U = Unknown. Click here for file
